# Polymorphism of *ORM1* Is Associated with the Pharmacokinetics of Telmisartan

**DOI:** 10.1371/journal.pone.0070341

**Published:** 2013-08-05

**Authors:** Wang-Qing Chen, Yan Shu, Qing Li, Lin-Yong Xu, Mary W. Roederer, Lan Fan, Lan-Xiang Wu, Fa-Zhong He, Jian-Quan Luo, Zhi-Rong Tan, Yi-Jing He, Hong-Hao Zhou, Xiang Chen, Wei Zhang

**Affiliations:** 1 Pharmacogenetics Research Institute, Institute of Clinical Pharmacology, Hunan Key laboratory of Pharmacogenetics, Central South University, Changsha, Hunan, P. R. C; 2 Department of Pharmaceutical Sciences, School of Pharmacy, University of Maryland, Baltimore, Maryland, United States of America; 3 Institute of Pharmacogenomics and Individualized Therapy, UNC Eshelman School of Pharmacy, Chapel Hill, North Carolina, United States of America; College of Pharmacy, University of Florida, United States of America

## Abstract

**Background:**

The pharmacokinetics (PKs) and pharmacodynamics (PDs) of telmisartan varies among the individuals, and the main causes remain unknown. The aim of this study was to evaluate the impact of *ORM1*, as well as *ABCC2, ABCB1, ABCG2* and *SLCO1B3* polymorphisms, on the disposition of the drug and BP change after taking 40 mg telmisartan in 48 healthy Chinese males.

**Method:**

A total of 48 healthy males were included in this trial. Every volunteer ingested a single dose of 40 mg telmisartan, and the plasma drug concentration and blood pressure (BP) were measured up to 48 h.

**Result:**

In this study, the area under the plasma concentration-time curve (AUC) in the heterozygotes of *ORM1* 113AG was higher than that in the wild-type homozygotes, AUC_(0–48)_ (113AA vs. 113AG, 1,549.18±859.84 ng·h/ml vs. 2,313.54±1,257.71 ng·h/ml, *P* = 0.033), AUC_(0–∞)_ (113AA vs. 113AG, 1,753.13±1,060.60 ng·h/ml vs. 2,686.90±1,401.87 ng·h/ml, *P* = 0.016), and the change(%) of the diastolic blood pressure (DBP) from the baseline BP value also showed a significant difference between the *ORM1* 113AG and 113AA genotypes at 5 h after taking telmisartan (*P* = 0.026). This study also showed that the allele of *ABCC2* C3972T would affected the disposition of telmsiartan and the DBP change significantly after taking the drug. However, the common SNPs of *ABCG2* C421, *ABCB1* C3435T, and *SLCO1B3* T334G showed no impacts on the PKs of telmisartan or BP change(%) in our trial.

**Conclusion:**

The *ORM1* A113G polymorphism was associated with the PKs variability after taking telmsiartan, as well as *ABCC2* C3972T. The heterozygotes of *ORM1* 113AG showed a larger AUC and a notable BP change(%) from the baseline compared with the wild-type.

**Trial Registration:**

Chinese Clinical Trial Registry ChiCTR-TNC-10000898

## Introduction

There are about 1 billion hypertension patients worldwide and the number is still sharply increasing. Effective use the antihypertensive agents has become a particular challenge for the public health, as the blood pressure (BP)-lowering responses is diverse and hard to predict in individuals [1]. Telmisartan is a highly selective antagonist for the angiotensin II receptor type 1, and has the unique pharmacological properties characterized by its long duration of action and increased the tolerability. Telmisartan is equivalent to ramipril in the patients with vascular disease or high-risk diabetes occurred and associated with less angioedema [2].

In the healthy volunteers and hypertensive patients, a remarkable inter-individual variability had been reported with the peak concentration in plasma (C_max_) and the area under the plasma concentration-time curve (AUC) of telmisartan [3]–[6]. Furthermore, it has been reported that after a high dose of telmisartan, the drug was eliminated faster in Caucasian than in Chinese [4]–[5], suggesting that an “ethnic” difference was existed in the drug’s disposition with unknown mechanism, while another population pharmacokinetics study of telmisartan suggested that the “race difference” was not large [6]. There were a few studies focused on the pharmacogenetics of telmisartan, however, the discoveries contributed little to the variability of pharmacokinetics (PKs). Telmisartan was not metabolized by the cytochrome P450 system, and almost all of the drug was excreted as the unchanged parent drug into the feces [7]. Thus, the variability of the pharmacokinetics (PKs) of telmisartan may occur in the absorption, distribution, or excretion processes.

When administrated orally, telmisartan shows an absolute bioavailability of 43%, a long elimination half-life of almost 24 h, and more than 99.5% of telmisartan binds to the plasma proteins [8]. Telmisartan displays a saturable binding to the alpha-1-acid glycoprotein 1 (AAG1; encoded by *ORM1*) in addition to the human serum albumin [9]. The AAG is a major binding protein in plasma for various basic drugs, and is encoded by 2 loci, i.e., *ORM1* and *ORM2,* and the content of *ORM1* is dominant. Unlike *ORM2*, *ORM1* is highly polymorphic [1]. There are 3 common haplotypes present at the *ORM1* locus (ORM1*F1 has 113A/520G; ORM1*F2 has 113A/520A and ORM1*S has 113G/520G) [10], [12]. Recent investigations suggested that the ORM1 *S genotypes had a stronger binding force to the drugs, such as quinidine, disopyramide and nortriptyline, thus the *S alleles may decreased the concentrations of the free drugs in the serum [11], [13]. And it’s reported that the allele of 520A was rare in Asia [10], [11], and the minor allele frequency (MAF) is 0.03% in Asia (data from hapmap), which supported that the haplotype of *F2 may also rare [12],[15]. Meanwhile, our institute previous researches found that the alleles of *ORM1* A113G can significantly affect the disposition of the drugs, nortriptyline and warfarin (data wasn’t published).

The multidrug-resistance protein 2 (MRP2, encoded by *ABCC2*), is a member of the ATP-binding cassette transporters family and plays a dominant role in the transport of various drugs, including telmisartan [6], [17]–[18]. *ABCC2* was highly polymorphic, and it was reported that three common SNPs C-24T, G1249A and C3972T [19], [25] were in linkage disequilibrium and likes to decrease the transporter function probably by a posttranscriptional modification on transporter protein expression [19], [20]. It was reported that *ABCC2* C-24T altered the pharmacokinetics of various drugs, including irinotecan, SN-38 and mycophenolic acid [21], [22]. However, some other studies found that *ABCC2* C-24T polymorphism can’t alter the drug’s transport [26]. Besides MRP2, some other transporters also participated in the disposition of telmisartan, such as the multiple drug resistance-1 protein (MDR1, encoded by *ABCB1*), including the human breast cancer resistance protein 2 (BCRP2, encoded by *ABCG2)*, and the influx pump of the human organic anion transporting polypeptide 1B3 (OATP1B3, encoded by *SLCO1B3*) [16], [27]–[30]. For MDR1, the polymorphism C3435T on exon 26 as a silent mutation, would affect the expression level and transfer functions [18], even affect the concentration of the substrates *via* absorption or excretion. It also has been suggested that the *ABCG2* C421A may affect the protein expression, membrane surface translocation, efflux activity, or ATPase activity [24]. It was reported that the patients with C421A mutation achieved a higher plasma drug levels, suggesting a significant reduction in BCRP2 mediated excretion [23],even affected the drug’s response [24]. And the previous reports had shown that the common polymorphisms T334G on *SLCO1B3* may alter the transporter’s activity in *vitro* and *vivo* [31], then alter the pharmacokinetic profile of the drugs, such as mycophenolic acid, rosuvastatin [31].

In this study, we intend to investigate the impacts of the polymorphisms of *ORM1*, *ABCC2*, *ABCB1*, *ABCG2*, and *SLCO1B3* on the PKs and the BP change after taking the drug.

## Methods

The protocol for this trial and supporting TREND checklist are available as supporting information; see Checklist S1 and Protocol S1.

### Subjects and Study Design

A total of 48 unrelated Chinese healthy males from Changsha city were enrolled in this clinical trial between May 2010 and Dec 2010. The subjects were excluded if they had a history or evidence of hepatic, renal, gastrointestinal, or hematologic abnormalities; any other acute or chronic disease; or an allergy to any drug. No medications, herbal medicines, alcohol, citrus juices, or beverages containing caffeine were permitted during the study. The mean age of the subjects was 22.83±2.51 y (range 19∼34 y), the mean weight was 63.33±7.75 kg (range 49∼78 kg), and the mean height was 171.57±5.22 cm (range 162∼183 cm) (Table 1, Table S1).

**Table 1:** The demographics information of the participants.

Number	Gender	Age(y)	Height (cm)	Weight (kg)	BMI(kg/m^2^)	Baseline SBP (mmHg)	Baseline DBP (mmHg)
48	male	22.83±2.51(19.00, 34.00)	171.57±5.22(162.00, 183.00)	63.33±7.75(49.00, 78.00)	21.73±2.09(17.57, 26.89)	115.00±11.53(95.00, 145.00)	77.04±8.39(60.00, 100.00)

BMI, body mass index.

The study protocol was approved by the Ethical Committee of the Institute of Clinical Pharmacology, Central South University. The registration number (ChiCTR-TNC-10000898) and the trial protocol were validated in the Chinese Clinical Trial Registry. The trial was carried out in the institute of Clinical Pharmacology, Changsha, Hunan, China. Written informed consent and the clinical characteristics were obtained from each subjects before the trial. Each of the volunteers ingested a single tablet of a 40 mg dose of telmisartan (Micardis; Boehringer Ingelheim International GmbH, Ingelheim, Germany) with 200 mL water at 08∶00. The subjects received a standardized breakfast 2 h after the administration of telmisartan, and received a warm meal at 12∶00 and supper at 18∶00. Five milliliters of the venous blood samples were drawn into EDTA tubes at 0 (before administration), 0.25, 0.5, 0.75, 1, 1.25, 1.5, 2, 3, 4, 6, 8, 10, 12, 24, and 48 h after administration. The sitting BP of each participants were measured twice using a mercury manometer at 0, 1, 2, 3, 4, 5, 6, 7, 8, 9, 10, 11, 12, 24, 48 h after administration. The plasma was separated by centrifugation at 3000×*g* for 15 min and stored at −20°C until extraction and analysis. The DNA was extracted through the standard phenol-chloroform extraction method.

### PKs and BP Change(%) from the Baseline

The PKs of telmisartan were characterized by C_max_, T_max_, T_1/2_, AUC_(0–48)_, and AUC_(0–∞)_. The elimination rate constant (k_e_) was determined by linear regression analysis of the log-linear part of the elimination phase of the concentration-time curve. The T_1/2_ was calculated by the equation T_1/2_ =  ln2/k_e_. The AUC_(0–∞)_ was calculated by the combination of the linear and log-linear trapezoidal rules, with extrapolation to infinity by division of the last measured concentration by k_e_. The CL/F was calculated with the quotation CL/F = Pxo/AUC_(0–∞)_, where Pxo represents the administration dose [15]; [16]. The BP change (%) from the baseline was calculated using the equation (BP - BP_0_)/BP_0_×100%, which BP_0_ represents the BP before the drug administration.

### Genotyping

The alleles of *ORM1* A113G were determined by a PCR-RFLP procedure as described below. The primers for *ORM1* A113G were 5′-GAACTGAATCTATGTTTGTCTTCC-3′ (Forward) and 5′-CGACCACAGCCAGCAGGG-3′ (Reversed) and the endonuclease enzyme was *EconI*. For there is a high degree of homology between *ORM1* and *OMR2*, then we took the PCR product sequencing, and verified that the PCR products were belong to *ORM1* but not *ORM2* gene. *ABCC2* C-24T SNPs was detected by pyro-sequencing (Pyromark Q96 ID System, Qiagen, Germany). The forward primer was 5′-GGCAAGGTTAACGATTAAATGG-3′; the reverse primer was 5′-GCAGAACTTCTCCAGCATGAT-3′ (with 5′-biotin), and the sequencing primer was 5′-TCATATTAATAGAAGAGTCT-3′. We determined the *ABCG2* C421A alleles by direct sequencing. The forward primer was 5′-ACTGCAGGTTCATCATCATTAGCTAGA-3′, and the reverse primer was 5′-CCGTTCGTTTTTTTCATGATTC-3′. Genotyping the *ABCB1* C3435T as the method reported [33]. The genotypes of *ABCC2* C3972T, *ABCC2* G1249A, and, *SLCO1B3* T334G were determined by a PCR-RFLP procedure as described in previous reports. The primers for *ABCC2* C3972T were 5′-GTGGACTGTTCGGCTGAGTT-3′ (Forward) and 5′-TCACTCCACCTACCTTCTCCATG-3′ (Reversed) and the endonuclease enzyme was *Bsh*1285I. The primers for *ABCC2* G1249A were 5′-GGGCAAAGAAGTGTGTGGAT-3′ (Forward) and 5′-TGGGATTACAAGCACCATCA-3′ (Reversed) and the endonuclease enzyme was *Nco*I. The primers for *SLCO1B3* T334G were 5′-GAAGGTACAATGTCTTGGGC-3′ (Forward) and 5′-CTCTCAAAAGGTAACTGCCC-3′ (Reversed) and the endonuclease enzyme was *Alu* I.

### The Linkage Disequilibrium Analysis

The linkage disequilibrium analysis of the *ABCC2* variants (C-24T, G1249A and C3972T) was constructed using the online software SHEsis (http://analysis2.bio-x.cn/my Analysis.php).

### Measurement of the Plasma AAG1 level

The concentration of AAG1 was determined using a commercial ELISA kit (ab108852, ABCAM, Cambridge, UK) for the quantitative determination according to the instruction by the manufacturer. The detection linearity of this kit was from 3.13 to 200 ng/ml. The plasma samples were diluted 1∶25000 with the diluted buffer. Absorbance values were read at 450 nm on the multimode ELISA reader (Beckman Coulter, Miami, FL, USA).

### Determining the Concentration of Telmisartan in Plasma

A Waters Alliance 2695 liquid chromatographic system (Waters, Milford, MA, USA) equipped with a HyPurity C18 column (2.1 mm×150 mm, 5 µm; Thermo Hypersil-Keystone, America) was used to determine the concentration of telmisartan in the plasma. The column temperature was 40°C. The mobile phase consisted of acetonitrile and 5 mM ammonium acetate (containing 0.1% acetic acid) at a ratio of 45∶55 (v: v), and the flow rate was 0.3 mL/min. Irbesartan (100 ng/mL) was used as the internal standard. Fifty microliters of irbesartan and 400 µL of acetonitrile were added into 180 µL of plasma. After mixing on a vortex mixer for 5 min and waiting for 10 min, the mixture was then centrifuged at 15000 rpm for 5 min. A total of 200 µL of supernatant was put into an injection tube, and an aliquot of 5 µL of supernatant in tubes was injected into the HPLC system each time. Mass spectrum (MS) detection was performed on a Waters Quattro Micro API mass spectrometer (Waters, Milford, MA, USA). For telmisartan and irbesartan, the precursor-to-production reactions monitored were m/z 515 to 429 and m/z 428 to 207, respectively. The range of quantification for telmisartan in the plasma was 5 ng/mL to 1000 ng/mL, and the inter-day and intraday precisions for all analyses were less than 15% (coefficient of variation).

### Statistical Analyses

All results were expressed as mean ± SD in the text and tables and, for clarity, as mean ± SEM in the figures. Statistical significance of the difference was evaluated using SPSS statistical software (SPSS Inc., ver.18.0). A *P* value of <0.05 was considered to be statistically significant. The contribution of the multiple factors to the variability of the parameters was evaluated using the stepwise linear regression analysis, and those variables with significant *P* value in the models were shown. The χ^2^ test was used to test whether the distribution met the Hardy-Weinberg equilibrium. Statistical comparison of the PKs parameters including C_max_, AUC_(0–48)_, and AUC_(0–∞)_ among non-carriers, heterozygous, and homozygous carriers was done by ANOVA after data points were naturally log-transformed, while CL/F was analyzed directly through ANOVA. When the *P* value was significant, a Tukey HSD test was followed to analysis the distribution between each two groups. The distribution of the T_max_ and T_1/2_, among the different genotypes were analyzed by the Kruskal-Wallis test. Repeated-measure ANOVA (genotypes×time) with Greenhouse-Geisser correction, and the Tukey tests for post-hoc multiple comparisons were used to compare the BP change(%) from the baseline BP value.

## Results

### Genotyping

Of these 48 subjects, we detected the polymorphisms of *ORM1* A113G and the genotypes of *ABCC2*, *ABCB1, ABCG2*, and *SLCO1B3*, and the frequencies of each genotype was shown in Table 2. The distributions of all the alleles in this population met the Hardy-Weinberg equilibrium. The linkage disequilibrium among the *ABCC2* single nucleotide polymorphisms (SNPs) was analyzed, and the results showed that the alleles of *ABCC2* C3972T was mostly in a linkage with *ABCC2* C-24T (D′  = 0.997, r^2^ = 0.031), and the D′ value was 0.793 and r^2^ = 0.606 when the analysis tested between the SNPs of *ABCC2* G1249A and C-24T.

**Table 2:** Summary of Genetic Variations of SNPs in this Study.

Genes	RS No.	Nucleotide substitution	Location	Amino Acids	MAF^a^ (CHB)	MAF^a^ (ASW)	MAF^a^ (CEU)	MAF^b^ (CHB)
*ORM1*	rs17650	A113G	Exon	Gln/Arg	0.275[37]	N/A	N/A	0.208
*ABCC2*	rs717620	C-24T	5′UTR	–	0.201	0.035	0.181	0.281
	rs3740066	C3972T	Exon	Ile/Ile	0.267	0.331	N/A	0.250
	rs2273697	G1249A	Exon	Val/Ile	0.106	0.243	0.132	0.083
*ABCB1*	rs1045642	C3435T	Exon	Ile/Ile	0.374	0.205	0.571	0.375
*SLCO1B3*	rs4149117	T334G	Exon	Ala/Ser	0.266	0.518	0.143	0.354
*ABCG2*	rs2231142	C421A	Exon	Gln/Lys	0.292	0.044	0.111	0.219

a data published on hapmap; b, data calculated in this study, N = 48; N/A, no data found in hapmap.

MAF: Minor Allele frequencies; CHB: Han Chinese in Beijing, China; ASW: African ancestry in Southwest USA; CEU: Utah residents with Northern and Western European ancestry from the CEPH collection *ORM1*, orosomucoid 1; *ABCC2*, ATP-binding cassette, sub-family C, member 2; *ABCG2*, ATP-binding cassette, sub-family G, member 2; *ABCB1,* ATP-binding cassette, sub-family B, member 1; *SLCO1B3,* solute carrier organic anion transporter family, member 1B3; SNP, single nucleotide polymorphisms; AUC_(0–48)_ the area under the plasma concentration-time curve (AUC) from 0 to 48 h; AUC_(0–∞),_ AUC from 0 to ∞; C_max_, the peak concentration in plasma; CL/F, clearance; T_1/2_, elimination half-life; T_max_, the time to C_max._

### Telmisartan PKs and BP Change(%) from the Baseline

**Figure 1:**
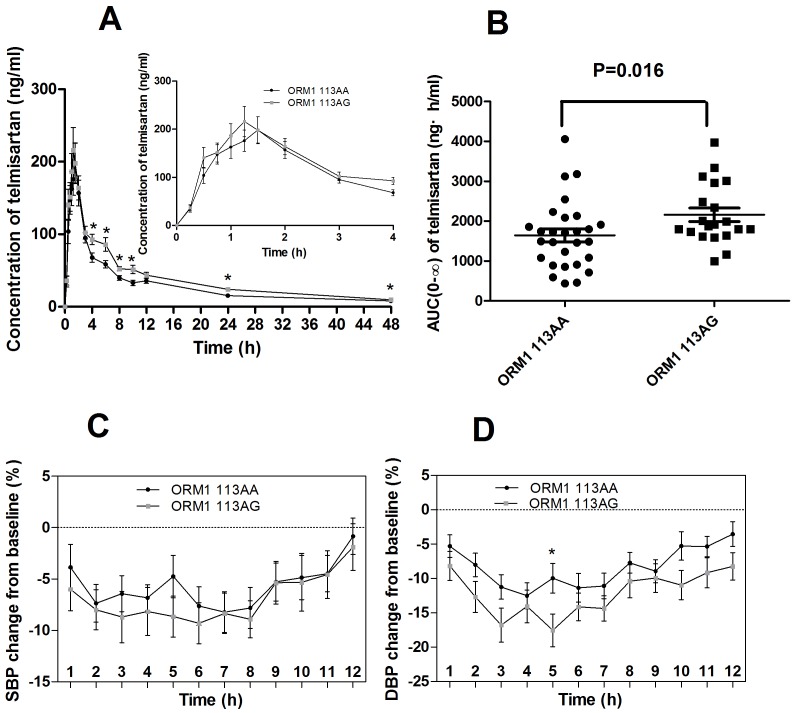
The impacts of *ORM1* genotypes on the PKs and BP change(%) after 40 mg telmisartan. A, Concentration; B, AUC_(0–∞)_; C, SBP change(%) from the baseline; D, DBP change(%) from the baseline.

**Figure 2:**
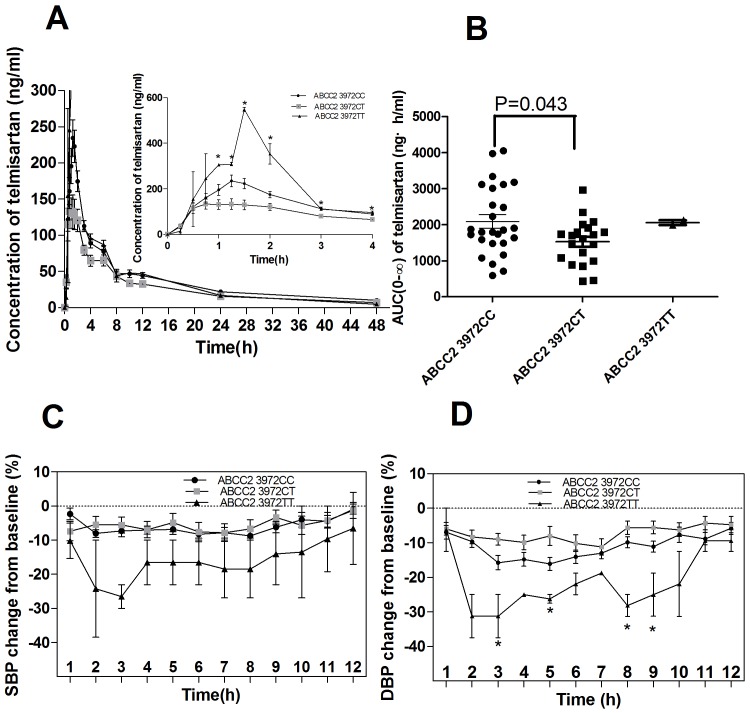
The impacts of *ABCC2* genotypes on the PKs and BP change(%) after 40 mg telmisartan. A, Concentration; B, AUC_(0–∞)_; C, SBP change(%) from the baseline; D, DBP change(%) from the baseline.

After an oral dose of 40 mg telmisartan, the mean AUC from 0 to 48 h (AUC_(0–48)_) in 48 healthy males was 1,793.75±1,065.94 ng·h/mL, the AUC from 0 to infinity (AUC_(0–∞)_) was 2,048.23±1,251.07 ng·h/mL, the C_max_ was 2,61.15±165.42 ng/mL, time to C_max_ (T_max_) was 1.28±0.54 h, elimination half-life (T_1/2_) was 17.15±8.83 h, and the clearance (CL/F) was 27.88±19.10 L/h. The PK parameters of telmisartan varied significantly in inter-individuals, for example, the AUC_(0–48)_, AUC_(0–∞)_, C_max_, T_max_, T_1/2_, and CL/F of telmisartan varied 3.9-, 14.5-, 17.8-, 6.0-, 7.6-, and 14.5-folds, respectively. After taking 40 mg telmisartan, the BP values of the participants declined lasted for more than 12 h, compared with the basal BP values. The decline degree of the BP was more notable in DBP than SBP, and the most significantly declined DBP occurred at 3∼9 h (Table 3, Figure 1, 2) when measured the BP change(%) from the baseline up to 48 h.

**Table 3:** Impacts of different SNPs on BP change (%) from the baseline after an oral administration of 40 mg telmisartan in 48 health males.

BP	Genes	Genotypes	Basal BPvalue (0 h)	Change (%) from the baseline 3 h	Change(%) from the baseline 4 h	Change(%) from the baseline 5 h	Change (%) from the baseline 6 h	Change(%) from the baseline 7 h	Change(%) from the baseline 8 h	Change(%) from the baseline 9 h	Change(%) from the baseline 10 h
DBP	*ORM1*(A113G)	AA(28)	75.75±7.20	11.23±9.48	12.52±9.98	9.96±11.37	11.35±11.06	11.08±9.77	7.72±8.08	8.94±8.86	5.26±10.99
		AG(20)	78.85±9.74	16.79±11.06	14.07±10.68	17.55±10.65	14.14±8.94	14.36±8.13	10.40±10.77	9.95±9.29	10.96±9.50
		*P* value	0.211	0.074	0.564	0.026*	0.418	0.182	0.364	0.564	0.061
SBP	*ORM1*(A113G)	AA(28)	113.96±10.76	6.42±9.26	6.82±6.71	4.75±10.84	7.63±9.89	8.22±10.52	7.80±10.57	5.28±9.72	4.85±11.44
		AG(20)	116.45±12.68	8.70±11.11	8.16±10.44	8.65±8.92	9.30±8.88	8.34±8.78	8.91±8.06	5.35±9.31	5.32±12.54
		*P* value	0.468	0.373	0.355	0.180	0.253	0.916	0.818	0.950	0.967
DBP	*ABCC2*(C3972T)	CC (26)	79.15±8.96	15.72±10.55	14.79±10.23	16.07±9.78	14.03±9.90	12.95±8.36	9.82±8.00	11.09±7.98	7.64±11.39
		CT (20)	74.00±7.21	8.95±7.54	9.88±9.46	7.98±12.19	10.11±10.87	11.16±10.49	5.64±8.76	5.56±8.10	6.20±8.85
		TT (2)	80.00±0.00	31.25±8.84	25.00±0.00	26.25±1.77	16.88±2.65	18.75±0.00	28.13±4.42	25.00±8.84	21.88±13.26
		*P* value	0.103	0.005*	0.069	0.013*	0.445	0.394	0.026*	0.010*	0.203
SBP	*ABCC2*(C3972T)	CC (26)	115.96±11.96	7.30±8.47	7.04±5.91	6.84±7.64	8.25±5.93	7.83±7.96	8.71±6.54	6.18±8.69	3.99±10.87
		CT (20)	113.75±10.69	5.55±10.52	6.90±10.69	4.75±12.67	7.60±12.69	7.82±11.49	6.67±12.12	3.31±10.00	5.58±12.64
		TT (2)	115.00±21.21	26.54±4.90	16.54±9.25	16.54±9.25	16.54±9.25	18.46±11.97	18.46±11.97	14.04±12.78	13.46±19.04
		*P* value	0.819	0.062	0.290	0.241	0.346	0.298	0.317	0.350	0.719

Data were shown as mean±SD; *P<0.05; **P<0.01.

*ORM1*, orosomucoid 1; *ABCC2*, ATP-binding cassette, sub-family C, member 2; BP, blood pressure; SBP, systolic blood pressure; DBP, diastolic blood pressure.

### The Plasma AAG1 Concentration Level

There was no significant difference of the plasma AAG1 concentration in different *ORM1* genotypes (*P* = 0.801), the concentration of AAG1 in individual with *ORM1* 113AA was 444.25±112.86 ng/ml (range 264.17∼700.83 ng/ml), and the concentration in *ORM1* 113AG was 452.92±133.05 ng/ml (range 240.83∼738.33 ng/ml).

### Association between the *ORM1* Genotypes and Telmisartan PKs and BP Change(%) from the Baseline

As shown in Figure 1 and Table 4, the subjects with *ORM1* 113AG had a higher AUC compared with the wild-type *ORM1* 113AA genotype, AUC_(0–48)_ (AA vs. AG, 1,549.18±859.84 vs. 2,313.54±1,257.71 ng·h/ml, *P* = 0.033), AUC_(0–∞)_ (AA vs. AG, 1,753.13±1,060.60 vs. 2,686.90±1,401.87 ng·h/ml, *P* = 0.016). The results of the repeated measure ANOVA which corrected by Greenhouse-Geisser showed that the *ORM1* A113G alleles had no significant impacts on the BP change(%) from the baseline after taking 40 mg telmisartan. While we found that the subjects with the genotype of *ORM1* 113AG appeared to have a more apparent BP change compared with the wildtype, although the *P* value was not significant except the DBP change(%) 5 h (*P* = 0.026) after administration (Figure 1, Table 3). There was no significant difference in the C_max_, CL/F, and T_1/2_ between the *ORM1* genotypes after taking 40 mg telmisartan (Table 4).

**Table 4:** Telmisartan PKs of 48 health males with different genotypes.

Gene	SNPs	Genotype	AUC_(0–48)_(ng·h/ml)	AUC_(0–∞)_ (ng·h/ml)	C_max_(ng/ml)	CL/F(l/h)	T_1/2_ (h)
*ORM1*	A113G	AA (28)	1,549.18±859.84	1,753.13±1,060.60	246.87±156.93	32.38±21.43	15.63±5.09
		AG (20)	2,313.54±1,257.71	2,686.90±1,401.87	293.00±184.06	18.63±8.74	20.01±12.81
		*P* VALUE	0.033*	0.016*	0.289	0.355	0.113
*ABCC2*	C3972T	CC (26)	1,871.18±817.89	2,090.83±952.69	313.32±172.05	18.80±10.32	15.83±4.11
		CT (20)	1,289.89±506.60	1,526.47±626.97	176.48±106.47	10.59±6.39	20.09±13.27
		TT (2)	1,973.90±135.44	2,057.50±97.27	548.22±12.93	32.89±0.78	12.26±3.11
		*P* VALUE	0.031*	0.105	0.001**	0.001**	0.321
		CC vs. CT	0.035*	0.105	0.005**	0.008**	0.476
		CC vs.TT	0.896	0.964	0.243	0.083	0.237
		CT vs. TT	0.311	0.526	0.014*	0.004**	0.147
*ABCC2*	C-24T	CC (24)	1,809.42±843.05	2,017.04±976.09	306.16±182.01	18.37±10.92	15.61±4.19
		CT (21)	1,348.93±525.05	1,588.64±637.52	191.52±112.96	25.10±10.75	19.98±12.95
		TT (3)	2,214.17±427.04	2,411.88±617.65	467.57±139.99	17.24±3.89	14.62±4.65
		*P* VALUE	0.060	0.161	0.008**	0.005**	0.523
*ABCC2*	G1249A	GG(41)	1597.79±667.24	1832.01±790.09	256.45±156.87	15.39±9.41	18.01±9.84
		GA(6)+AA(1)	1998.44±1108.08	1232.57±992.12	355.01±236.12	21.30±14.17	14.09±4.01
		*P* VALUE	0.285	0.417	0.296	0.296	0.614
*ABCG2*	C421A	CC (30)	1,579.22±713.71	1,800.86±797.05	258.14±165.95	15.49±9.96	17.69±10.97
		CA (15)	1,679.29±719.04	1,840.07±798.27	297.90±170.29	17.87±10.22	16.34±11.36
		AA (3)	1,943.43±921.94	2,459.68±1,394.14	186.58±133.13	19.57±8.94	20.75±5.44
		*P* Value	0.669	0.573	0.481	0.540	0.265
*ABCB1*	C3435T	CC (20)	1,606.83±646.33	1,791.71±814.66	237.23±126.55	14.23±7.59	15.48±5.05
		CT (20)	1,673.53±789.53	1,920.07±849.29	291.52±206.17	17.49±12.37	18.95±12.29
		TT (8)	1,598.62±913.64	1,846.30±1,026.48	274.70±165.31	16.48±9.92	18.67±8.88
		*P* VALUE	0.587	0.317	0.951	0.984	0.076
*SLCO1B3*	T334G	GG (18)	1,702.25±740.60	1,877.33±806.34	298.07±204.22	17.88±12.25	15.90±4.08
		GT (26)	1,574.47±755.06	1,829.07±896.92	232.81±132.75	13.97±7.97	18.56±11.78
		TT (4)	1,704.89±797.20	1,914.58±939.86	338.56±196.27	25.38±13.32	17.31±8.43
		*P* VALUE	0.793	0.929	0.424	0.307	0.994

Data were shown as mean±SD;

* *P*<0.05; **P<0.01.

PKs, pharmacokinetics; *ORM1*, orosomucoid 1; *ABCC2*, ATP-binding cassette, sub-family C, member 2; *ABCG2*, ATP-binding cassette, sub-family G, member 2; *ABCB1,* ATP-binding cassette, sub-family B, member 1; *SLCO1B3,* solute carrier organic anion transporter family, member 1B3; SNP, single nucleotide polymorphisms; AUC_(0–48)_ the area under the plasma concentration-time curve (AUC) from 0 to 48 h; AUC_(0–∞),_ AUC from 0 to ∞; C_max_, the peak concentration in plasma; CL/F, clearance; T_1/2_, elimination half-life; T_max_, the time to C_max._

### Association between the Transporters Gene Polymorphisms and Telmisartan PKs and BP Change(%) from the Baseline

The parameters of AUC_(0–48)_, C_max_, CL/F in the different *ABCC2* C3972T genotypes varied significantly (*P* = 0.031, 0.001, and 0.001, respectively) (Figure 2, Table 4); The repeated measure ANOVA results showed that *ABCC2* C3972T polymorphism would significantly affected the DBP change(%) from the baseline after taking telmisartan(*P* = 0.018) (Table 5). The DBP change(%) from the baseline varied significantly at 3, 5, 8, 9 h (*P* = 0.05, 0.013, 0.026 and 0.010 respectively) among the ABCC2 C3972T genotypes(Figure 2, Table 3). The C_max_ and CL/F of the different *ABCC2* C-24T genotypes also manifested a remarkable difference (*P* = 0.008 and 0.005, respectively)(Table 4). In this study, the polymorphisms of *ABCC2*(G1249A), *ABCB1* (C3435T), *SLCO1B3* (T334G) and *ABCG2* (C421A) had no significant influence on the PKs of telmisartan and the BP change(%) from the baseline after taking telmisartan (Table 3, 4, 5).

**Table 5:** The significance affected by time or genotypes on the BP change(%) from the baseline.

	SBP change (%) from the baseline				DBP change (%) from the baseline			
	Mauchly’s	Time	Time*genotype	genotype	Mauchly’s	Time	Time*genotype	genotype
ORM1 A113G	0.004	0.000	0.748	0.770	0.000	0.000	0.258	0.096
ABCC2 C3972T	0.006	0.000	0.262	0.162	0.000	0.000	0.067	0.018*
ABCC2 G1249A	0.005	0.000	0.795	0.370	0.000	0.030	0.975	0.144
SLCO1B3 T334G	0.001	0.000	0.842	0.995	0.000	0.000	0.431	0.634
ABCG2 C421A	0.001	0.000	0.300	0.255	0.000	0.000	0.451	0.896
ABCB1 C3435T	0.001	0.000	0.369	0.375	0.000	0.000	0.372	0.241

* *P*<0.05;

*ORM1*, orosomucoid 1; *ABCC2*, ATP-binding cassette, sub-family C, member 2; SBP, systolic blood pressure; DBP, diastolic blood pressure; Time, the time point in this study including 1, 2, 3, 4, 5, 6, 7, 8, 9, 10, 11, 12, 24, 48 h after taking telmisartan.

### Stepwise Regression Analysis

The stepwise multiple regression analysis results showed that the AUC variability of telmisartan was affected by the polymorphisms of *ORM1* A113G (*P* = 0.028) and *ABCC2* C3972T (*P* = 0.031). In this stepwise multiple regression models, we correct the multiple factors Age, BMI, and basal BP values. The genetic polymorphisms *ORM1* A113G*ABCC2 C3972T contributed R^2^ = 0.150 and 0.153 respectively to AUC_(0–48)_ and AUC_(0–∞)._ The *ORM1* A113G*ABCG2 C421A contributed R^2^ = 0.160 to the DBP 5 h change(%) from the baseline. There was no genetic variants contributed significantly to Cmax, CL/F, T_1/2_ and SBP 5 h change (%) from the baseline (Table 6).

**Table 6:** The P valve of variable factors on PK and BP change parameters in the corrected models.

Independents	AUC_(0–48)_	AUC_(0–∞)_	5 h DBP change(%) from the baseline
*ORM1* A113G	0.028*	0.018*	0.012*
*MRP2* C3972T	0.031*	0.045*	–
*ABCG2* C421A	–	–	0.099
*ABCB1* C3435T	–	–	–
*SLCO1B3* T334G	–	–	–
R^2^	0.150	0.153	0.160
Ajusted R^2^	0.113	0.115	0.122

Data were shown as mean±SD;

* *P*<0.05;

** *P*<0.01.

*ORM1*, orosomucoid 1; *ABCC2*, ATP-binding cassette, sub-family C, member 2; *ABCG2*, ATP-binding cassette, sub-family G, member 2; *ABCB1,* ATP-binding cassette, sub-family B, member 1; *SLCO1B3,* solute carrier organic anion transporter family, member 1B3; AUC_(0–∞)_ the area under the plasma concentration-time curve(AUC) from 0 to ∞; C_max_, the peak concentration in plasma; CL/F, clearance; T_1/2_, elimination half-life; T_max_, the time to C_max_; SBP, systolic blood pressure; DBP, diastolic blood pressure.

## Discussion

In this study, we for the first time reported that the polymorphism of *ORM1* A113G was associated with the variability of the drug AUC and BP change(%) from the baseline after taking 40 mg telmisartan in Chinese healthy males. The content of the plasma AAG1 had no significant difference between *ORM1* genotypes, while the heterozygotes of *ORM1* 113AG demonstrated a higher AUC of the drug and a apparent BP decline after taking 40 mg telmisartan. This result was consistent with the previous reports that the *ORM1* variants can affect the disposition of the drug, alter the concentrations of the free drugs in the serum [11]–[14]. It was reported that there were 7 drug-protein binding sites with different affinities on the AAG surface, and more binding sites on the AAG surface with *ORM1* 113G than *ORM1* 113A, thus the affinity was much stronger in the former haplotype [2], [32]. The more drugs bind to plasma proteins, the fewer drugs would be excreted; and more stored bound-drugs would transformed into the plasma free drug. Thus the total plasma drug concentration and AUC in *ORM1* 113AG heterozygote would be remains in a higher level. We can speculate that when the drug was in a high plasma concentration for a long duration of time, the substrate would manifest as a stronger pharmacodynamics. In our study, when compared with the wild-type *ORM1* 113AA, the *ORM1* 113AG genotype decrease the basal BP more markedly at 5 h after taking the drug (SBP, *P* value = 0.180; DBP, *P* value = 0.026). The same tendency of BP change(%) from the baseline between 113AA and 113AG genotype was also presented in the other time points, although the *P* value were not significant (Table 3). The difference of BP change between the *ORM1* genotypes was not as significant as the PKs, the reasons may be there were many physical, condition or mental factors would influence the BP. In this research, the significant difference of drug concentration between *ORM1* genotypes were in 1∼4 h after the drug administration, while the most significant difference of DBP change(%) from the baseline occurred in 3∼9 h. It seemed that the BP change was lagged behind the PKs several hours. We speculated that because the *ORM1* mostly affected the distribution phase of the drugs rather than the other disposition phase. So the significant difference caused by *ORM1* polymorphisms may occurred in an earlier period of drug disposition(1–4 h, in Figure 1), and the previous researches found that after administering 3 h later, the BP started to decline, so the BP change seemed to be lagged.

The difference of BP change(%) from the baseline among *ABCC2 C3972T* three genotypes was manifested in a concentration-dependent fashion. After administering 40 mg telmisartan, the heterozygotes of *ABCC2* 39732CT showed the lowest AUC, C_max_, and displayed the minimum SBP and DBP change(%) from the baseline, while the genotype TT manifested as the highest AUC, C_max_, and the maximum change of the BP. The significant difference of DBP change among *ABCC2* 3972 genotypes occurred at 3, 5, 8 and 9 h after taking telmisartan(Table 3). For *ABCC2* C3972 and C-24T in a strong linkage disequilibrium, our research was consistent to the previous reports that the C_max_ of telmisartan in the −24CT genotype group was significantly greater than that in the −24CC genotype (*P* = 0.0094) in Japanese renal transplant patients (3). While, the impacts of *ABCC2* C3972T polymorphism on PKs and BP chang(%) from the baseline were not consistent with the gene-dose effect, the possible reasons may be two. On the one hand, as *ABCC2* C-24T, C3972T, and G1249A SNPs were in a strong linkage disequilibrium, thus the other two SNPs might interfere with C3972T to affect the variability of PKs/PDs. It has been reported that the MRP2 transport function may be affected by the *ABCC2* polymorphisms in a haplotype-dependent model(33). And besides this three SNPs, some unknown SNPs may also participated in the disposition of telmisartan and may interacted with *ABCC2* C3972T, which need a further research. On the other hand, only 2 subjects carried the genotype of ABCC2 3972TT participated in this study, thus the parameters of this two subjects may won’t reflect the population characteristics of the homozygote group. This results need confirmed in a more large population.

The underlying mechanisms of telmisartan PKs and PDs highly varied among the individuals still remain unknown. There were a few pharmacogenomics studies on telmisartan’s disposition, while the research results contributed little to the variability [34]. Some other studies reported that genetic factors, such as *ABCB1* C3435T, *SLCO1B3* T334G and other related polymorphisms, showed no influence on the PKs variability [6],[34]–[36]. In our trial, we confirmed that the polymorphisms of *SLCO1B3* T334G, *ABCB1* C3435T, *ABCG2* G421A had no significant influence on telmisartan disposition. It was reported that the haplotype of UGT1A3*4a can influence the telmisartan’s PKs [35]. The previous reports also showed that CL/F of telmisartan was associated with age, dose, gender, alcohol consumption, race and liver function [6], but in our study the factor of age was nonfunctional. It was also manifested in our study that the DBP decline was stronger than SBP after taking telmisartan (Table 3), which implied that telmisartan would be an ideal choice for the hypertensive with a higher DBP value.

In our study, all the frequencies of the 7 SNPs in the 48 participates was consistent with the reported frequencies in CHB population. As reported in the literatures, Mastana found that there was a significant clinical decrease of the ORM1*S allele frequency from West to East (r = 0.80, P<0.01) in 48 populations, and the frequency of the allele ORM1*S was lowering from 31% to 19% when presented in central Asia towards the Southeast [38]. The ORM1*S allele frequency in the present study was within these ranges among the populations of Asia. Different populations may possess various genetic backgrounds, which would cause the variability of PKs or even PDs of the substrates. As shown in Table 2, the MAF of *ABCC2* C3972T and *G1249A*, *ABCB1* C3435T, *SLCO1B3* T334G almost be similar in three main populations. While the frequencies of the alleles *ABCC2* -24T, *ABCG2* 421G shown a apparent decrease in ASW population. Thus, we speculated that the PKs of Telmisartan in West may differ from Asia, which was supported the conclusion that after a high dose of telmisartan, the drug was eliminated faster in Caucasian than in Chinese subjects [4]–[5].

However, some shortcomings existed in our study. First, the subjects in our trial were young and healthy males, with well self-regulation ability on the BP fluctuations and their body’s regulation ability may conceal the BP change to some degree. Second, for the BP value was fluctuated in a day, and the time showed a significant impacts on the BP change(%) from the baseline(Table 5). So in this study, there was no enough data to value the actual antihypertensive pharmacodynamics among the genotypes if just compare the BP change *via* took the pre-dose BP as baseline merely. We should monitor the volunteer’s blood pressure at the same time on another day when taking no telmisartan, in order to remove the diurnal variation within individuals. Third, the number of participants was not large enough to get a convincing conclusion. For example, there was no subject with homozygous *ORM1* 113GG genotypes enrolled in this trial for us to explore the influence of the genotype 113GG. Hence, this conclusion needs to be identified in a larger population in the further research, especially in the hypertensive.

To explore the possible reasons for the metabolism variation, can benefit us to improve the efficiency of telmisartan. Our study showed that the polymorphisms of *ORM1* A113G can absolutely affected the disposition of telmisartan *in vivo* after administration, as well as the SNPs of *ABCC2* C3972T. It is a new discovery would help us to understanding the mechanism of the noteworthy disposition difference after telmisartan taken, however it need further researches to confirm in a large scale population.

## Supporting Information

Table S1 **The clinical characteristics data and baseline BP values of the 48 subjects.**
(DOC)

Checklist S1 **The TREND Statement Checklist.**
(DOC)

Protocol S1 **The Clinical Trial Protocol on the pharmacokinetics of telmisartan in Chinese males.**
(DOC)
