# Supplementary material for: Polymorphism of ORM1 Is Associated with the Pharmacokinetics of Telmisartan
Source: PLoS One. 2013 Aug 5;8(8):e70341. doi: 10.1371/journal.pone.0070341 (PMC3734062; doi:10.1371/journal.pone.0070341)
Supplement: Table S1 — The clinical characteristics data and baseline BP values of the 48 subjects. (DOC) [file pone.0070341.s001.doc]

**Supplement Table S1 The clinical characteristics data and baseline BP values of the 48 subjects**

| Serial Number | Gender | Age  (y) | Height（cm） | Weight  (kg) | BMI  (kg/m2) | Baseline SBP(mmHg) | Baseline DBP (mmHg) |
| --- | --- | --- | --- | --- | --- | --- | --- |
| 1 | male | 27 | 166 | 60 | 21.77 | 120 | 78 |
| 2 | male | 25 | 182 | 70 | 21.13 | 118 | 70 |
| 3 | male | 25 | 167 | 55 | 19.72 | 116 | 70 |
| 5 | male | 24 | 170 | 75 | 25.95 | 120 | 80 |
| 6 | male | 23 | 174 | 64 | 21.14 | 110 | 70 |
| 7 | male | 23 | 167 | 60 | 21.51 | 120 | 90 |
| 8 | male | 25 | 174 | 61 | 20.15 | 105 | 65 |
| 9 | male | 24 | 175 | 68 | 22.20 | 105 | 70 |
| 10 | male | 23 | 171 | 57 | 19.49 | 105 | 70 |
| 11 | male | 22 | 180 | 73 | 22.53 | 135 | 85 |
| 12 | male | 24 | 162 | 53 | 20.20 | 100 | 65 |
| 13 | male | 23 | 172 | 59 | 19.94 | 130 | 90 |
| 14 | male | 25 | 168 | 60 | 21.26 | 105 | 80 |
| 15 | male | 25 | 170 | 56 | 19.38 | 110 | 80 |
| 16 | male | 24 | 176 | 60 | 19.37 | 110 | 80 |
| 18 | male | 20 | 172 | 74 | 25.01 | 145 | 100 |
| 19 | male | 23 | 165 | 60 | 22.04 | 105 | 80 |
| 20 | male | 22 | 167 | 75 | 26.89 | 110 | 80 |
| 21 | male | 21 | 175 | 75 | 24.49 | 130 | 85 |
| 22 | male | 22 | 165 | 56 | 20.57 | 105 | 75 |
| 23 | male | 24 | 168 | 56 | 19.84 | 105 | 85 |
| 24 | male | 21 | 162 | 54 | 20.58 | 114 | 72 |
| 25 | male | 21 | 172 | 70 | 23.66 | 124 | 80 |
| 26 | male | 22 | 165 | 68 | 24.98 | 114 | 80 |
| 27 | male | 20 | 172 | 60 | 20.28 | 105 | 65 |
| 28 | male | 24 | 170 | 64 | 22.15 | 100 | 70 |
| 29 | male | 23 | 173 | 65 | 21.72 | 95 | 65 |
| 30 | male | 19 | 183 | 78 | 23.29 | 120 | 80 |
| 31 | male | 22 | 178 | 78 | 24.62 | 120 | 80 |
| 32 | male | 21 | 175 | 70 | 22.86 | 120 | 90 |
| 33 | male | 21 | 179 | 64 | 19.97 | 110 | 70 |
| 35 | male | 22 | 178 | 74 | 23.36 | 120 | 80 |
| 36 | male | 34 | 170 | 52 | 17.99 | 110 | 70 |
| 37 | male | 21 | 166 | 58 | 21.05 | 140 | 70 |
| 38 | male | 21 | 167 | 49 | 17.57 | 100 | 70 |
| 39 | male | 20 | 173 | 75 | 25.06 | 110 | 80 |
| 40 | male | 22 | 163 | 59 | 22.21 | 110 | 70 |
| 41 | male | 21 | 164 | 58 | 21.56 | 110 | 80 |
| 42 | male | 23 | 176 | 68 | 21.95 | 110 | 80 |
| 43 | male | 21 | 170 | 60 | 20.76 | 130 | 80 |
| 44 | male | 24 | 170 | 70 | 24.22 | 124 | 84 |
| 45 | male | 33 | 179 | 73 | 22.78 | 110 | 60 |
| 46 | male | 21 | 172 | 66 | 22.31 | 120 | 77 |
| 47 | male | 21 | 178 | 68 | 21.46 | 122 | 72 |
| 48 | male | 24 | 166 | 54 | 19.60 | 105 | 60 |
| 49 | male | 20 | 175 | 63 | 20.57 | 130 | 80 |
| 50 | male | 21 | 177 | 58 | 18.51 | 100 | 80 |
| 51 | male | 22 | 173 | 71 | 23.72 | 138 | 96 |
