# Supplementary material for: Polymorphism of ORM1 Is Associated with the Pharmacokinetics of Telmisartan
Source: PLoS One. 2013 Aug 5;8(8):e70341. doi: 10.1371/journal.pone.0070341 (PMC3734062; doi:10.1371/journal.pone.0070341)
Supplement: Checklist S1 — The TREND Statement Checklist. (DOC) [file pone.0070341.s002.doc]

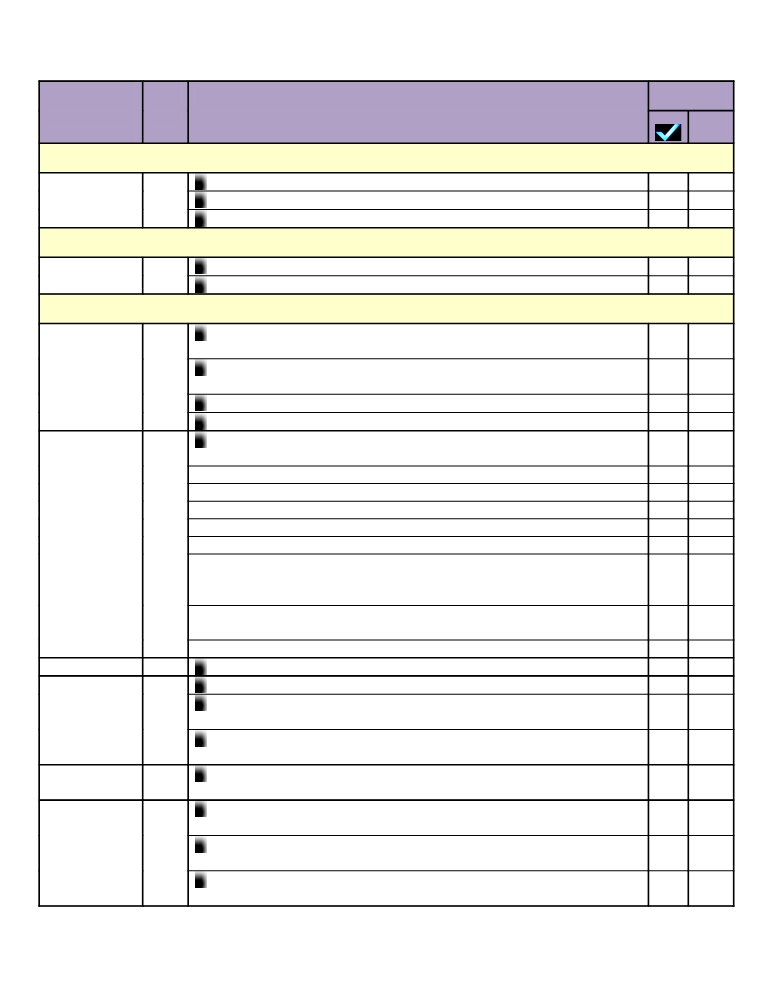
o\

TREND Statement Checklist

Paper Item Descriptor Reported?

Section/ No

Title and Abstract

Title and 1

Abstract

Introduction

Background 2

Methods

Participants 3

Interventions 4

Objectives 5

Outcomes 6

Sample Size 7

Assignment 8

Method

Information on how unit were allocated to interventions √ Title

Structured abstract recommended √ Abstract

Information on target population or study sample √ Abstract

Scientific background and explanation of rationale √ Introduction

Theories used in designing behavioral interventions √ Introduction

Eligibility criteria for participants, including criteria at different levels in √ Method recruitment/sampling plan (e.g., cities, clinics, subjects)
Method of recruitment (e.g., referral, self-selection), including the
sampling method if a systematic sampling plan was implemented
Recruitment setting √ Method

Settings and locations where the data were collected √ Method

Details of the interventions intended for each study condition and how

and when they were actually administered, specifically including:
 o Content: what was given? √ Method

o Delivery method: how was the content given? √ Method

o Unit of delivery: how were the subjects grouped during delivery?

o Deliverer: who delivered the intervention?

o Setting: where was the intervention delivered? √ Method

o Exposure quantity and duration: how many sessions or episodes or

events were intended to be delivered? How long were they

intended to last? √ Method

o Time span: how long was it intended to take to deliver the

intervention to each unit? √ Method

o Activities to increase compliance or adherence (e.g., incentives)

Specific objectives and hypotheses √ Introduction

Clearly defined primary and secondary outcome measures √ Method
Methods used to collect data and any methods used to enhance the

quality of measurements √ Method

Information on validated instruments such as psychometric and biometric

properties √ Method

How sample size was determined and, when applicable, explanation of any

interim analyses and stopping rules √ Method

Unit of assignment (the unit being assigned to study condition, e.g., i

ndividual, group, community) Same Assignment

Method used to assign units to study conditions, including details of any restriction (e.g., blocking, stratification, minimization)

Inclusion of aspects employed to help minimize potential bias induced due to non-randomization (e.g., matching)


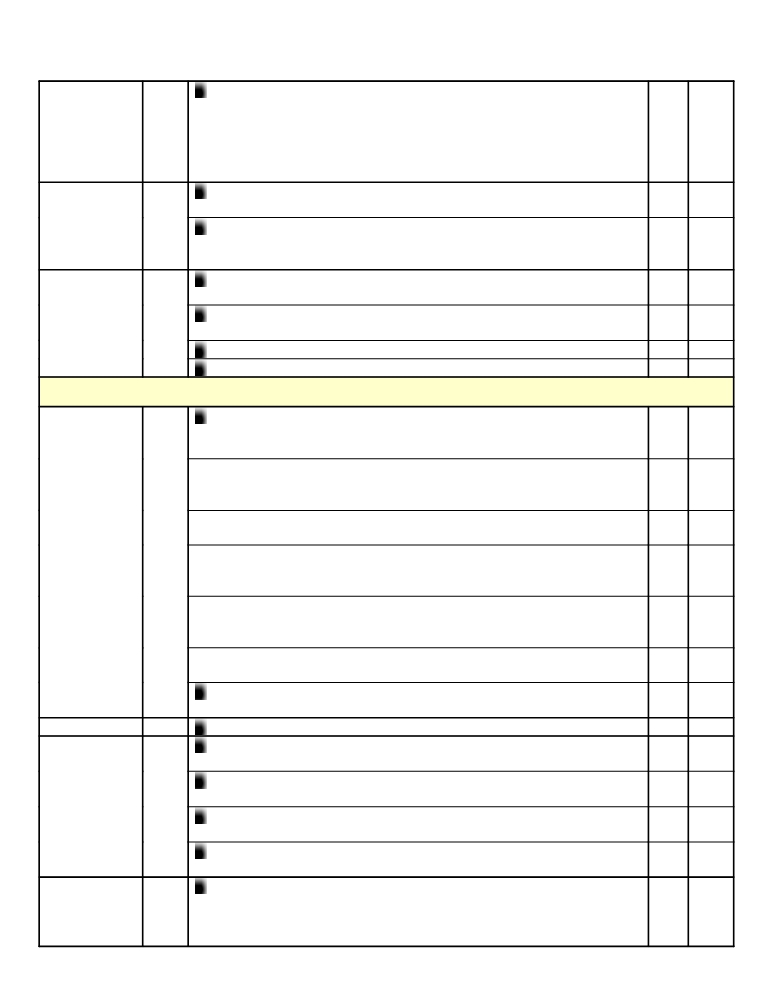

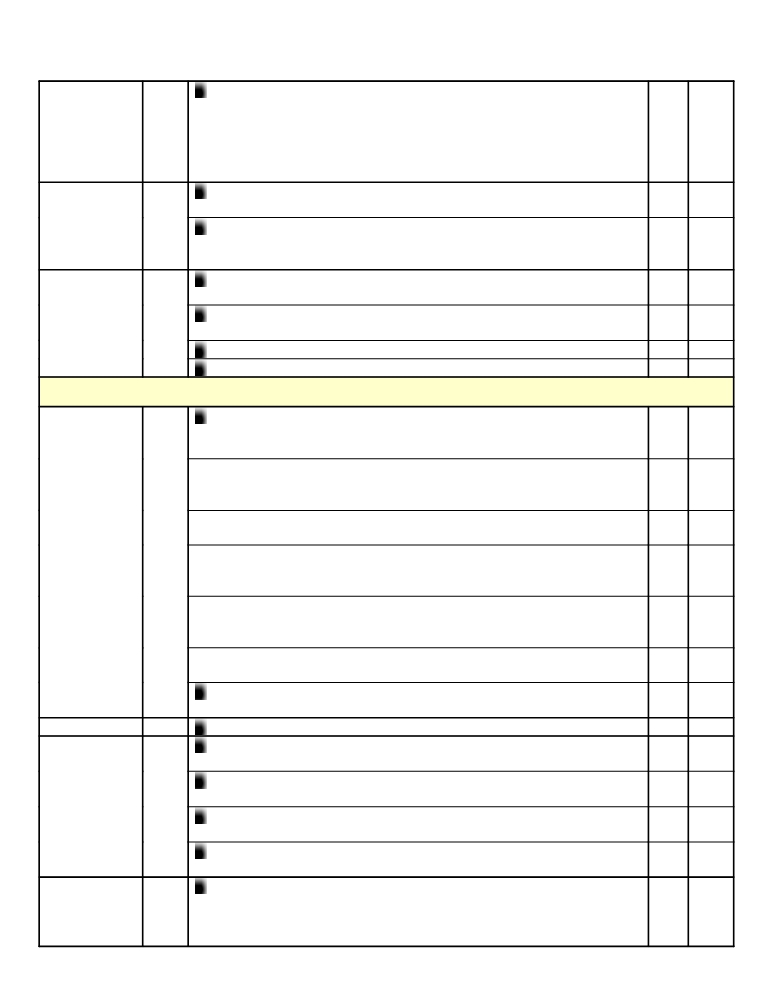


TREND Statement Checklist

Blinding 9

(masking)

Unit of Analysis 10

Statistical 11

Methods

Results

Participant flow 12

Recruitment 13

Baseline Data 14

Baseline 15

equivalence

Whether or not participants, those administering the interventions, and

those assessing the outcomes were blinded to study condition assignment;

if so, statement regarding how the blinding was accomplished and how it was

assessed. No Blinding

Description of the smallest unit that is being analyzed to assess
intervention effects (e.g., individual, group, or community) See Protocol
If the unit of analysis differs from the unit of assignment, the analytical method used to account for this (e.g., adjusting the standard error
estimates by the design effect or using multilevel analysis) See Protocol
Statistical methods used to compare study groups for primary methods outcome(s), including complex methods of correlated data √ Method Statistical methods used for additional analyses, such as a subgroup
analyses and adjusted analysis √ Method

Methods for imputing missing data, if used

Statistical software or programs used

Flow of participants through each stage of the study: enrollment, assignment, allocation, and intervention exposure, follow-up, analysis (a diagram is strongly recommended) See flow-diagram

o Enrollment: the numbers of participants screened for eligibility,

found to be eligible or not eligible, declined to be enrolled, and

enrolled in the study

o Assignment: the numbers of participants assigned to a study

condition

o Allocation and intervention exposure: the number of participants

assigned to each study condition and the number of participants

who received each intervention

o Follow-up: the number of participants who completed the follow-

up or did not complete the follow-up (i.e., lost to follow-up), by

study condition

o Analysis: the number of participants included in or excluded from

the main analysis, by study condition

Description of protocol deviations from study as planned, along with reasons

Dates defining the periods of recruitment and follow-up see protocol
Baseline demographic and clinical characteristics of participants in each

study condition see Supplement table

Baseline characteristics for each study condition relevant to specific see Supplement table

disease prevention research

Baseline comparisons of those lost to follow-up and those retained, overall and by study condition

Comparison between study population at baseline and target population of interest

Data on study group equivalence at baseline and statistical methods used to control for baseline differences


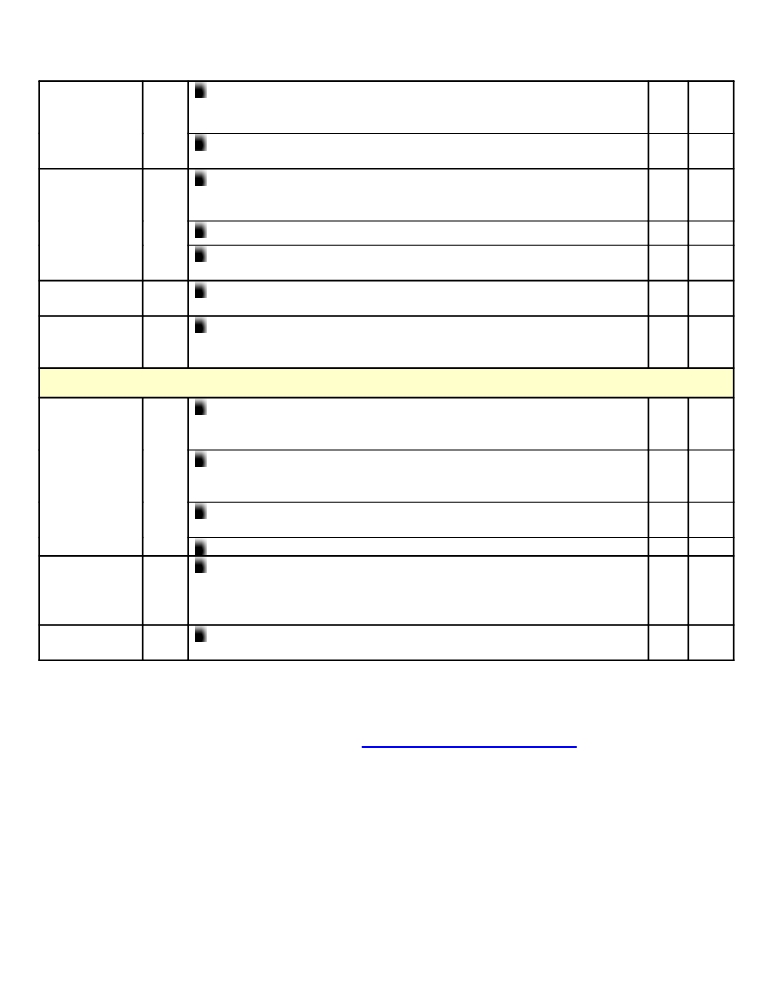


TREND Statement Checklist

Numbers 16 Number of participants (denominator) included in each analysis for each √ Results&Discussion

analyzed study condition, particularly when the denominators change for different

outcomes; statement of the results in absolute numbers when feasible

Indication of whether the analysis strategy was “intention to treat” or, if Yes

not, description of how non-compliers were treated in the analyses

Outcomes and 17 For each primary and secondary outcome, a summary of results for each

estimation estimation study condition, and the estimated effect size and a confidence

interval to indicate the precision √ Results

Inclusion of null and negative findings √ Results

Inclusion of results from testing pre-specified causal pathways through which the intervention was intended to operate, if any √ Results

Ancillary 18 Summary of other analyses performed, including subgroup or restricted

analyses analyses, indicating which are pre-specified or exploratory √ Results

Adverse events 19 Summary of all important adverse events or unintended effects in each No

study condition (including summary measures, effect size estimates, and confidence intervals)

DISCUSSION

Interpretation 20 Interpretation of the results, taking into account study hypotheses,

sources of potential bias, imprecision of measures, multiplicative analyses,

and other limitations or weaknesses of the study √ Discussion

Discussion of results taking into account the mechanism by which the

intervention was intended to work (causal pathways) or alternative

mechanisms or explanations √ Discussion

Discussion of the success of and barriers to implementing the intervention,

fidelity of implementation √ Discussion

Discussion of research, programmatic, or policy implications √ Discussion

Generalizability 21 Generalizability (external validity) of the trial findings, taking into account

the study population, the characteristics of the intervention, length of

follow-up, incentives, compliance rates, specific sites/settings involved in

the study, and other contextual issues √ Discussion

Overall 22 General interpretation of the results in the context of current evidence

Evidence and current theory √ Discussion

From: Des Jarlais, D. C., Lyles, C., Crepaz, N., & the Trend Group (2004). Improving the reporting quality of

nonrandomized evaluations of behavioral and public health interventions: The TREND statement. American Journal of
Public Health, 94, 361-366. For more information, visit: <http://www.cdc.gov/trendstatement/>
