# Supplementary material for: Polymorphism of ORM1 Is Associated with the Pharmacokinetics of Telmisartan
Source: PLoS One. 2013 Aug 5;8(8):e70341. doi: 10.1371/journal.pone.0070341 (PMC3734062; doi:10.1371/journal.pone.0070341)
Supplement: Protocol S1 — The Clinical Trial Protocol on the pharmacokinetics of telmisartan in Chinese males. (DOC) [file pone.0070341.s003.doc]

The Clinical Trial Protocol on the pharmacokinetics of telmisartan in Chinese males

Title: Polymorphism of ORM1 is Associated with the Pharmacokinetics of Telmisartan

Objectives of Study: To explore the influence of MRP2,MDR1 and OATP1B3 polymorphism on telmisartan’s pharmacokinetics and pharmacodynamics

Inclusion criteria: 1. Healthy men,aged between 19~40y, and the age years difference among the subjects is no more than 10y in one trial; 2. Weight over than 50kg,and in the scope of ±10％ideal weight; 3. Without gastrointestinal tracts diseases, liver or severely kidney injury; 4. Without supersensitive history on drugs,and without postural hypotension; 5. Take no medicine drugs in last two weeks before trials; 6. All subjects sign the written informed consent.

Exclusion criteria: 1. Systolic blood pressure <100mm Hg, heart rate<50/min or>100/min; 2. With the supersensitive history on drugs, and supersensitive with telmisartan; 3. Participated other clinical trails in last three months, or taken any other drugs in last two weeks; 4. With gastrointestinal tracts diseases, liver or severely kidney injury.

Study execute time: From 2010-5-1 to 2010-12-30

Sample size: As the guideline of the pharmacokinetic trials, each group should take 8-12 participant participated and the minor allele frequency of *ORM1* *F/*S was about 0.21 in Chinease Han population when search the pubmed. So we need more than 40 volunteers if we hope to enroll 12 subjects with the [heterozygote](app:ds:  heterozygote) of *OR M1* *F/*S. Total 48 Chinese Male participated.

Intervention：After an overnight fast, each of the volunteers ingested a single tablet of a 40-mg dose of telmisartan (Micardis; Boehringer Ingelheim International GmbH, Ingelheim, Germany) with 200 mL water at 08:00. The subjects received a standardized breakfast 2h after the administration of telmisartan, and they received a warm meal at 12:00 and supper at 18:00. Five milliliters of venous blood samples were drawn into EDTA tubes at 0 (before the administration), 0.25, 0.5, 0.75, 1, 1.25, 1.5, 2, 3, 4, 6, 8, 10, 12, 24, and 48h after administration. The sitting BP of each participant was measured twice using a mercury manometer at 0 h and each h after administration. The average BP was recorded.
